# Lung ultrasound score to predict development of acute chest syndrome in children with sickle cell disease

**DOI:** 10.1016/j.htct.2024.07.003

**Published:** 2024-09-23

**Authors:** Pedro P.M.G. Vieira, Josefina A.P. Braga, Rodrigo Regacini

**Affiliations:** Escola Paulista de Medicina da Universidade Federal de São Paulo (EPM UNIFESP), São Paulo, SP, Brazil

**Keywords:** Sickle cell disease, Acute chest syndrome, Lung ultrasound, Point of care ultrasound, Pediatrics, Children

## Abstract

**Objective:**

This study aims to identify lung ultrasound (LUS) findings associated with acute chest syndrome (ACS) at the time of admission and 24–48 h later, to compare these to chest radiography (CXR) findings and to establish a score to predict the development of this pulmonary complication in sickle cell disease (SCD) children

**Methods:**

A prospective observational study of SCD children presenting signs or symptoms of ACS evaluated by LUS and CXR at admission and 24–48 h later. A score was conceived to predict the evolution of ACS during hospitalization based on ultrasonographic findings.

**Results:**

Seventy-eight children were evaluated; 61 (78.2 %) developed ACS. A score greater than one at admission showed sensitivity, specificity, accuracy, and positive predictive value (PPV) of 75.4 %, 88.2 %, 78.2 %, and 95.8 %, respectively to predict ACS, while only 32 (52.5 %) CXR showed alterations. The development of ACS during hospitalization was unlikely for a score of zero and very likely for a score greater than one at admission. Regarding follow-up exams, a score greater than one showed sensitivity, specificity, accuracy, and PPV of 98.4 %, 76.5 %, 93.6 %, and 92.8 %, respectively to predict the development of ACS. ACS development was very unlikely for a score of zero and very likely for a score greater than zero in the follow-up.

**Conclusion:**

LUS is an effective tool to assess risk for the development of ACS in SCD children with clinical suspicion.

## Introduction

Sickle cell disease (SCD) is a group of autosomal recessive disorders characterized by mutations in the gene encoding the Beta chain of hemoglobin (Hb) with sickle cell anemia being the homozygous form of this mutation. Other types of SCD are determined by the presence of the Hb S mutation associated with another mutation in the gene encoding the beta- chain - Hb C, Hb D, and beta-thalassemia.

Hb S exhibits changes in the physicochemical characteristics of red blood cells, leading to sickling in situations of oxidative stress that cause the main acute crises of SCD, namely vaso-occlusive crises (VOC) and acute chest syndrome (ACS).

ACS was defined by Vichisnky et al. by the presence of at least one sign or symptom of lower airway infection such as cough, chest pain, fever, hypoxemia or tachypnea and the presence of a new pulmonary infiltrate by chest radiography (CXR) that is not related to atelectasis.^1^^,^^2^

ACS occurs as a complication in up to 50 % of VOC and is the main cause of death and the second leading cause of hospitalization among patients with SCD. Radiographic changes occur, on average, on the third day of hospitalization, which can delay ACS diagnosis and the beginning of its treatment.^3^

In the last decade, lung ultrasound (LUS) has been demonstrated to be a more sensitive and specific method than CXR in the diagnosis of pneumonia in the pediatric population ^4^^,^^5^ and, more recently, in the assessment of ACS.^6^, ^7^, ^8^, ^9^, ^10^, ^11^ LUS has a number of advantages compared to CXR, namely that it is free from ionizing radiation and can be performed at the bedside by the clinical team.^5^

This study aims to identify LUS findings associated with ACS at the time of admission and 24–48 h later, to compare these to CXR findings and to establish a score to predict the development of this pulmonary complication in pediatric patients.

## Methods

This is a prospective observational study undertaken in a quaternary hospital in Brazil from March 2020 to February 2021. It was approved by the institutional review board. All patients and their legal guardians gave their assent or consent. This research was conducted in accordance with the Helsinki Declaration as revised in 2008 and received no specific grant from any funding agency in the public, commercial or not-for-profit sectors.

The estimated sample size was 61 patients based on a LUS sensitivity of 98 %,^8^ a 50 % incidence of ACS in patients hospitalized with VOC^3^ and a confidence interval of 95 %, as suggested by Buderer.^12^

The inclusion criteria were previous diagnosis of SCD, age under 18 years, and the presence of any signs or symptoms suggestive of lower airways infection (cough, chest pain, fever, hypoxemia, or tachypnea).

The exclusion criteria were ACS in the previous six weeks,^13^ the inability to determine the development of ACS (e.g. transference to another service, study dropout or death), and the impossibility of performing CXR and LUS within 12 h and again within 24–48 h of the onset of signs or symptoms of lower airways infection.

The medical team responsible for treating the patients did not receive any information regarding the results found by the LUS and no patient had their treatment changed or influenced by this study.

Clinical and demographic data were recovered from the patient's files. The development of ACS for the porpoise of this study was defined by the presence of at least one sign or symptom of lower airways infection and the presence of consolidation or acinar opacities on any CXR performed during the whole hospitalization, even those performed after the 48 h established for the follow-up analysis. Atelectasis or peribronchial thickening were not considered relevant for ACS diagnosis.^1^^,^^2^

The CXR exams were analyzed by a pediatric radiologist, blinded to the patient's identity or the date of the exam. The presence of acinar opacities, consolidation and pleural effusion were recorded.^14^

All the CXR and LUS were performed within 12 h after inclusion of the patient in the study and also within 24–48 h of the first examination for patients who fulfilled the inclusion criteria for a follow-up evaluation.

All CXR exams were performed with a Siemens Axiom-Iconos R200 MD X-ray device in posteroanterior or anteroposterior views and all the LUSs were performed with a V-Scan dual probe GE device with a linear probe from 3.3 to 8 MHz in a transverse and longitudinal positions.

**Figure 1:**
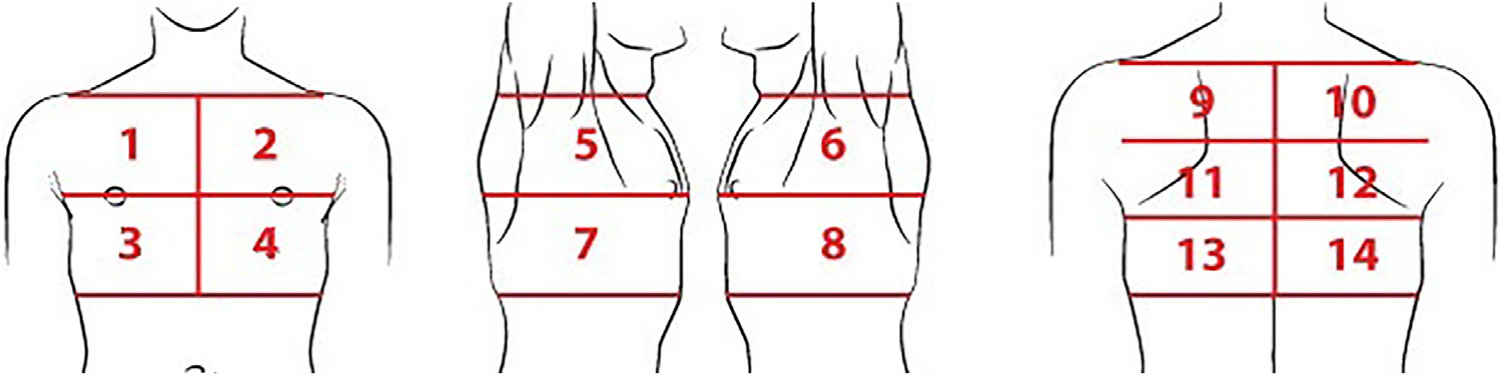
Lung areas to be scanned in each lung ultrasound exam. Regions 1 and 2: upper on the midclavicular line above the internipple line. Regions 3 and 4: basal on the midclavicular line below the internipple line. Regions 5 and 6: upper on the midaxillary line above the internipple line. Regions 7 and 8: basal on the midaxillary line below the internipple line. Regions 9 and 10: upper on the paravertebral line. Regions 11 and 12: middle on the paravertebral line. Regions 13 and 14: basal on the paravertebral line, above the curtain sign.

For the LUS, each hemithorax was divided into seven regions (Figure 1) as proposed by Soldati et al.^15^ All intercostal spaces were evaluated with patients in the sitting position and static images of the fourteen pre-defined regions were recorded. Dynamic images were also recorded when there was any finding that was better characterized by respiratory incursion and excursion. All LUS images were reviewed by the same radiologist who analyzed the CXRs, blinded to clinical data and radiographic findings.

All LUS exams were performed by an emergency pediatrician, who had received six hours of theoretical learning and practical training with 25 supervised exams completed, was blinded to the patient's radiological or clinical information.^16^

At the end of the procedure, each of the 14 pre-defined regions received a score according to the findings and the sum of the scores of each region defined the LUS score.^15^^,^^16^

**Figure 2:**
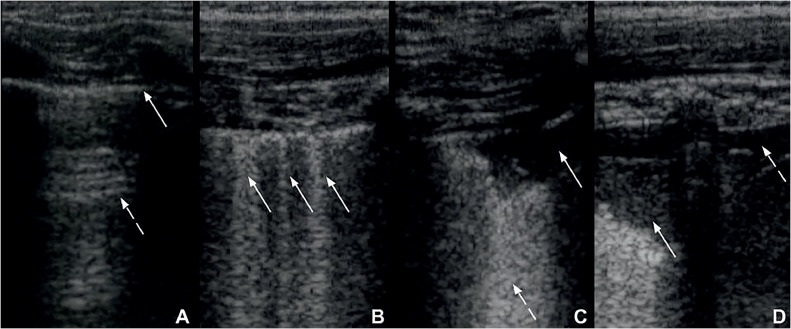
A. Lung ultrasound obtained with linear probe showing continuous and regular pleural line (continuous arrow) and presence of A-lines (dashed arrow), horizontal hyperechogenic artifacts due to the high reflectivity of the normally aerated lung. B: Lung ultrasound obtained with linear probe, showing presence of B-lines (continuous arrows), vertical white lines, due to alterations in the acoustical properties of the lung, representing the partial replacement of air in the alveolar space by fluid. C: Lung ultrasound obtained with linear probe, showing a small consolidation (continuous arrow), associated with hyperechogenic areas below (dashed arrow), representing the loss of aeration of lung parenchyma. D: Lung ultrasound obtained with linear probe, showing a larger consolidation (continuous arrow) and hyperechogenic area below, associated with a small pleural effusion (dashed arrow).
• One point: the pleural line is indented. Below the indent, vertical white lines (B-lines), due to local alterations in the acoustical properties of the lung are visible, representing the partial replacement of the air in the alveolar space by fluid (Figure 2B).
• Two points: the pleural line is broken. Below the breaking point, consolidated areas (hypoechogenic areas-subpleural consolidations) appear with associated hyperechogenic areas below the consolidated area (white lung – coalescent B-lines). This represents the loss of aeration and the transition of these areas toward acoustic properties like soft tissue over the entire area represented by the consolidation itself. Pleural effusion might or might not be identified (Figure 2C).
• Three points: the scanned area shows dense and largely extended white lung with or without larger consolidations. Pleural effusion might or might not be identified (Figure 2D).

• Zero points: the pleural line is continuous and regular. A-Lines (horizontal artifacts due to the high reflectivity of the normally aerated lung) are present (Figure 2A).

### Statistical analysis

Statistical tests were performed with a confidence interval of 95 % using Fisher's exact test or the chi-square test and described using odds ratio (OR), accuracy, sensitivity, specificity, positive predictive value (PPV) and negative predictive value (NPV).

The probability of developing ACS was classified according to the PPV as very unlikely (<10 %), unlikely (between 10 % and 33 %), uncertain (between 34 % and 66 %), likely (between 67 % and 90 %) and very likely (>90 %) as proposed by Lucey et al.^17^

Statistical analyses were performed using the software Statistical Package for Social Sciences (SPSS) for Windows version 22.0 (IBM Corp., Armonk, NY, USA).

## Results

A total of 112 patients met the inclusion criteria for the study, 34 of whom were excluded - one was transferred to another care unit, one had been diagnosed with ACS in the previous week, five were diagnosed with ACS on admission but the follow-up CXR was performed more than 48 h later and the follow-up CXR or LUS was not performed for 27 patients because they did not present lower airway signs or symptoms 48 h after admission. Sixteen were diagnosed with VOC at sites other than the chest, two with hemolysis crisis without other signs or symptoms, six with upper airway infections and three with urinary tract infections.

Thus, 78 patients with a median age of 11.2 years were included in the study: 67 (85.9 %) had sickle cell anemia, six (7.7 %) S-beta thalassemia, and five (6.4 %) SC hemoglobinopathy. Sixty-one (78.2 %) patients, with a mean age of 11.6 years (±4.4), developed ACS.

Among the 17 patients who did not develop ACS, ten were diagnosed with VOC at sites other than the chest and seven with upper airway infections without radiographic findings.

### Assessment of patients at the time of admission

The most common LUS findings at admission were pathological B-lines (46.2 %), coalescing B-lines (41.0 %), subpleural consolidations (33.3 %), pleural effusion (20.5 %), and consolidation (19.2 %). The LUS identified consolidation with sensitivity, specificity, and accuracy of 100 %, 88.3 % and 87.2 %, respectively using the CXR as the gold standard. The prevalence of each finding is listed in Table 1.

**Table 1:** Prevalence of lung ultrasound findings on admission associated with the final diagnosis of acute chest syndrome.

LUS finding	p-value	OR	Prevalence		
			Total (*n* = 78)	ACS (*n* = 61)	NACS (*n* = 17)
B-lines Coalescent B-lines Subpleural consolidation Consolidation Pleural effusion	0.117^a^ <0.001^b^ <0.001^b^ 0.032^b^ 0.017^b^	2.48 16.53 * * *	36/78 (46.2 %) 32/78 (41.0 %) 26/78 (33.3 %) 15/78 (19.2 %) 16/78 (20.5 %)	31/61 (50.8 %) 31/61 (50.8 %) 26/61 (42.6 %) 15/61 (24.6 %) 16/61 (26.2 %)	5/17 (29.4 %) 1/17 (5.9 %) 0/17 (0.0 %) 0/17 (0.0 %) 0/17 (0.0 %)

OR: odds ratio; LUS: lung ultrasound; ACS: acute chest syndrome; NACS: not diagnosed with acute chest syndrome.

⁎ Impossible to calculate odds ratio because all the subjects were diagnosed with acute chest syndrome.

a Chi-square test.

b Fisher's exact test.

There was a statistical association between the LUS performed at admission, the development of ACS and the presence of coalescent B-lines (p-value <0.001), subpleural consolidation (p-value <0.001), pleural effusion (p-value = 0.017) and consolidation (p-value = 0.032). All the patients presenting with subpleural consolidation, pleural effusion or consolidation at admission developed ACS.

The LUS score at admission was associated with the development of ACS (p-value <0.001) with a higher median in the group of subjects who would later be diagnosed with ACS than in the group of subjects who did not develop this condition during the study (2 versus 0; p-value <0.001).

The p-values, OR, accuracy, sensitivity, specificity, PPV and NPV for each score range for the admission LUS are listed in Table 1. The cutoff value of highest accuracy at admission (78.2 %) was a LUS score greater than one.

The probability of the development of ACS during hospitalization according to the LUS score at admission was unlikely for a score of zero, likely for a score of one and very likely for a score greater than one. It is noteworthy that a LUS score at admission greater than four presented a PPV of 100 % (Table 2).

**Table 2:** Lung ultrasound scores at admission and their association with the final diagnosis of acute chest syndrome.

LUS score	p-value	OR	Accuracy (%)	Sensitivity (%)	Specificity (%)	NPV (%)	PPV (%)	Probability of ACS
0 >0 >1 >2 >3 >4 >5	0.001^a^ 0.001^a^ <0.001^b^ <0.001^b^ <0.001^b^ <0.001^b^ <0.001^b^	0.01 7.5 23.0 32.8 28.4 * *	23.1 76.9 78.2 73.1 70.5 61.5 55.1	19.7 80.3 75.4 67.2 62.9 50.8 42.6	35.3 64.7 88.2 94.1 94.1 100.0 100.0	52.2 47.8 50.0 44.4 42.1 36.2 32.7	10.9 89.1 95.8 97.6 97.5 100.0 100.0	Unlikely Likely Very likely Very likely Very likely Very likely Very likely

OR: odds ratio; NPV: negative predictive value; PPV: predictive positive value; LUS: lung ultrasound; ACS: acute chest syndrome.

⁎ Impossible to calculate odds ratio because all the subjects were diagnosed with acute chest syndrome.

a Chi-square test.

b Fisher's exact test.

Of the 61 patients who developed ACS, only 32 (52.2 %) had abnormal CXR findings at the time of hospital admission.

### Assessment of patients 24–48 h after admission

In the evaluation performed 24–48 h after admission (follow-up), the most common LUS findings of the 78 patients were subpleural consolidation (56.4 %), coalescent B-lines (53.8 %), consolidation (50.5 %), pleural effusion (43.6 %) and B-lines (34.6 %). The prevalence of each finding is listed in Table 3.

**Table 3:** Prevalence of lung ultrasound findings on exams performed 24–48 h after admission associated with the development of acute chest syndrome.

LUS finding	p-value	OR	Prevalence		
			Total (*n* = 78)	ACS (*n* = 61)	NACS (*n* = 17)
B lines Coalescent B lines Subpleural consolidation Consolidation Pleural effusion	0.947^a^ <0.001^b^ <0.001^b^ <0.001^b^ 0.005^b^	0.96 * 9.57 * 8.28	27/78 (34.6 %) 42/78 (53.8 %) 44/78 (56.4 %) 39/78 (50.0 %) 34/78 (43.6 %)	21/61 (34.4 %) 42/61 (68.9 %) 44/61 (67.2 %) 39/61 (63.9 %) 32/61 (11.8 %)	6/17 (35.5 %) 0/17 (0.0 %) 3/17 (17.6 %) 0/17 (0.0 %) 2/17 (11.8 %)

OR: odds ratio; LUS: lung ultrasound; ACS: acute chest syndrome; NACS: not diagnosed with acute chest syndrome.

⁎ Impossible to calculate odds ratio because all the subjects were diagnosed with acute chest syndrome.

a Chi-square test.

b Fisher's exact test.

There was a statistical association between subpleural consolidation (p-value <0.001), coalescent B-lines (p-value <0.001), consolidation (p-value <0.001) and pleural effusion (p-value = 0.005). All the patients presenting with consolidation or coalescent B-lines developed ACS.

The follow-up LUS score was also associated with the development of ACS (p-value <0.001 - Table 4); a higher median was observed among the subjects who developed ACS (3.0 versus 0; p-value <0.001).

**Table 4:** Lung ultrasound scores 24–48 h after admission and their association with the final diagnosis of acute chest syndrome.

LUS score	p-value	OR	Accuracy (%)	Sensitivity (%)	Specificity (%)	NPV (%)	PPV (%)	Probability of ACS
0 >0 >1 >2 >3 >4 >5	<0.001^a^ <0.001^a^ <0.001^a^ <0.001^a^ <0.001^a^ <0.001^a^ <0.001^a^	0,00 144.0 195.0 46.8 36.1 30.2 *	7.7 92.3 93.6 92.3 87.2 82.1 78.2	1.6 98.4 98.4 95.1 86.9 78.7 72.1	29.4 70.6 76.5 82.4 88.2 94.1 100	7.7 92.3 92.9 82.4 65.2 55.2 50.0	7.7 92.393.8 95.1 96.4 98.0 100	Very unlikely Very likely Very likely Very likely Very likely Very likely Very likely

OR: odds ratio; NPV: negative predictive value; PPV: predictive positive value; LUS: lung ultrasound. ACS; acute chest syndrome.

⁎ Impossible to calculate odds ratio because all the subjects were diagnosed with acute chest syndrome.

a Fisher's exact test.

The p-values, OR, accuracy, sensitivity, specificity, PPV and NPV for each score range of the follow-up LUS are listed in Table 4. The cutoff value of highest accuracy at follow-up (9.6 %) was again for a LUS score greater than one (93.6 %).

The probability of the development of ACS during hospitalization according to the follow-up LUS score was very unlikely for a score of zero and very likely for a score greater than zero. A follow-up LUS score greater than five presented a PPV of 100 % (Table 4).

In comparison, 55 (90.2 %) of the 61 patients diagnosed with ACS during hospitalization had altered CXR during the follow-up.

## Discussion

ACS is the second leading cause of hospitalization and the main cause of death for children with SCD^2^; its diagnosis by CXR remains challenging due to the relatively late onset of radiographic findings.^3^ However, this study demonstrates that LUS can identify pulmonary findings associated with ACS development even at the time of admission, making it possible to determine which patients are at greater risk for ACS.

A meta-analysis of six studies published in 2022, five of which evaluated the pediatric population, found a LUS summary sensitivity of 92 % and summary specificity of 89 % for the diagnosis of ACS,^11^ similar to the results of this study.

In 2016, Colla et al.^7^ prospectively evaluated 20 adult patients with SCD during a VOC with the objective of evaluating early pulmonary abnormalities associated with ACS in LUS exams performed on arrival at the emergency department. Statistical associations of consolidation and pleural effusion with ACS were identified similar to this study. LUS pulmonary abnormalities were found in a mean time (7.4 h) earlier than the mean time for the CXR pulmonary abnormalities (35.9 h), suggesting the possibility of identifying ACS earlier by LUS.

A prospective study conducted by Daswani et al.^10^ with 91 pediatric patients diagnosed with SCD, who had fever in the 24 h prior to admission, evaluated LUS to identify consolidation and found a sensitivity of 85 % and a specificity of 95 %. Another prospective study conducted by Cohen et al.^6^, with the objective of determining the accuracy of LUS for the diagnosis of ACS due to the presence of consolidation, found sensitivity, specificity and accuracy of 88 %, 93 % and 92 %, resectively. The results described by these authors were similar to those of this study, with sensitivity, specificity, and accuracy of 100 %, 88.3 % and 87.2 %, respectively.

In this study, the development of ACS was very likely for LUS scores greater than one at both admission and in the follow-up exam, whereas a LUS score of zero makes the development of ACS unlikely at the time of admission and very unlikely 24–48 h later. No other study has evaluated the use of a LUS score to assess the risk of developing ACS.

The term ‘pulmonary infiltrate’ used by Vichinsky et al.^2^ to describe the radiographic alteration required to diagnose ACS does not have a corresponding definition in the current terms used in radiology. Thus, different pathophysiological processes, such as thickening of the interstitial space or an increase in the amount of alveolar fluid, can be mistakenly interpreted as pulmonary abnormalities related to ACS.^14^

The main pathophysiological component of the pulmonary parenchymal abnormalities in ACS is the increase in interstitial fluid due to vascular changes that occur because of acute hypoxia. Air in the alveolar space is partially^18^ (defined as acinar opacity on CXR and as ground glass on computed tomography) or completely (defined as consolidation in both diagnostic methods)^14^ replaced by pathological products. Therefore, in this study, only consolidation and acinar opacities were used for the diagnosis of ACS by CXR.

Although there is still no consensus regarding which specific sonographic changes are associated with the pathophysiological course of ACS, this study demonstrated a statistical association of ACS with coalescent B-lines, subpleural consolidations, consolidations, pleural effusion and the LUS score, both at admission and in the follow-up exam.

The LUS score has recently been demonstrated by Wang et al.^19^ to correlate lung function parameters in pediatric acute respiratory distress syndrome such as dynamic lung compliance, oxygenation index and duration of mechanical ventilation. It was also demonstrated by Alencar et al.^20^ that the LUS score can be a predictor of death, need for intensive care and endotracheal intubation in adult patients diagnosed with COVID-19. Both studies, although not describing disease-specific lesions, found an association between ultrasound scores and clinical outcome.

In this study, only one patient diagnosed with ACS had a follow-up LUS score of zero and follow-up CXR with central acinar opacity. The LUS performed on the third day showed a score of one and on the fourth day, a score of three. An early imaging test might not show any findings at first, even with a future development of ACS as this condition may be due to an infectious or embolic event or arise as a complication of a VOC^3^. Therefore, a normal initial LUS may appear as a false negative result, reinforcing the importance of follow-up exams in patients with clinical suspicion of ACS.

Furthermore, LUS may miss central lesions, which might also explain false negative results and reaffirm that CXR remains an important diagnostic tool in this population. Further studies are necessary to determine whether LUS alone will be able to evaluate pediatric patients with signs and symptoms of ACS and to diagnose this pulmonary complication. In the meantime, we believe that LUS can be used for the bedside evaluation of this population in addition to CXR.

A prospective study conducted by Preto-Zamperlini et al.,^8^ which enrolled 39 pediatric patients, evaluated the identification of consolidations by LUS performed at the time of admission and found sensitivity of 100 % and specificity of 76 %. The hypothesis put forward by the researchers was that LUS can identify smaller consolidations than those identified by CXR, which may have produced false positive results. Additionally, some abnormalities found by LUS may be due to previous lung injuries. Thus, performing LUS in asymptomatic patients can improve the diagnostic capacity of this method with a better knowledge of chronic lesions in each patient, even though the number of false positives was small in this study.

It is still uncertain whether any clinical intervention in patients identified as being at high risk for developing ACS would have any benefit. Thus, more studies are necessary to determine the role of LUS in this population. However, we believe that the ability to identify patients at increased risk of developing ACS, even with normal CXR, makes LUS a valuable diagnostic tool with the possibility of becoming a better diagnostic method than CXR.

Furthermore, as studies on the use of LUS as a diagnostic method for ACS are still scarce, the use of a score to stratify the risk of developing this complication can be a starting point to define which patients should be treated even without radiographic changes.

### Limitations

The main limitations of the current study are the lack of follow-up after discharge to assess whether the ultrasound findings were due only to previous injuries and the number of patients who were excluded because they had not undergone follow-up exams within the predetermined interval.

### Strengths

A sample of adequate size was evaluated. The LUS score and the technique of performing this exam are quick to learn and the results were achieved by an emergency pediatrician with the use of a portable ultrasound device, which is relatively low cost, and easy to handle and clean.

## Conclusion

LUS is an effective tool for the evaluation of patients with signs or symptoms of lower airway infections as it is able to identify findings associated to the development of ACS earlier than CXR. The proposed LUS score can identify patients at increased risk of developing ACS using a cutoff value of one.

## Funding

All authors declare no support from any organization for the submitted work

## Conflicts of interest

no financial relationships with any organizations that might have an interest in the submitted work in the previous three years; no other relationships or activities that could appear to have influenced the submitted work.
